# 

**Published:** 1895-12-21

**Authors:** 


**Notice to Advertisers and Subscribers.**

All Advertisements, Orders for Copies of The Hospital and Business Communications should be addressed to **THE MANAGER, (Not to The Editor)**, The Hospital, 428, Strand, London, W.C.

The Cost of Subscription, post free, is as follows:—Per week, 2½d.; per quarter, 2s. 8d.; per half-year, 5s. 3d.; per annum, 10s. 6d. Foreign:—Per Annum, 15s. 2d.; payable, in all cases, in advance.

### NOTICE TO CORRESPONDENTS.

All MS., Letters, Books for Review, and other matters intended for the Editor should be addressed The Editor, The Lodge, Porchester Square, London, W.

The Editor cannot undertake to return rejected MS., even when accompanied by stamped directed envelope.

**"Burdett's Hospital Annual," 1895.**

Sixth year of publication. 950 pp. Scarlet cloth, gilt lettered. Price 5s. A most complete guide to British, Colonial, Indian, and American Hospitals, Dispensaries, Nursing and Convalescent Institutions, and Asylums. A few complete sets of the preceding five issues of the "Annual" may still be obtained on application to the Publishers, The Scientific Press (Limited), 428, Strand, London, W.C.

**NOTICE.—Vol. XVIII. of "The Hospital" (April to September, 1895), in handsome cloth binding, is now on sale at the Office of this Journal. Price 7s. 6d. Cases for binding the Numbers contained in this Volume, 1s. 6d. Index to Vol. XVIII., 2½d., post free.**

**The Monthly Part for December is now ready, price 6d., by post, 8d.**

**IMPORTANT NOTICE.**

**On account of the Christmas Holidays, we beg to announce that we shall go to press with our issue of the 28th INSTANT on MONDAY NIGHT, the 23rd INST. Advertisements intended for insertion in this issue, should therefore reach us BY NOON ON MONDAY, the 23rd inst.**

### Scraps and Gleanings.

A new crematorium has just been opened at Glasgow.

There are over 2,000 deaf mutes scattered throughout London.

The project is afoot for the provision of a seawater bath for the metropolis.

During the five years, 1890-1894, 1,120 persons were killed by lightning in the United States.

Her Majesty has ordered a supply of cast linen to be forwarded to Charing Cross Hospital, also to University College Hospital, of which Her Majesty is patron.

There are no fewer than 1,284 practising physicians of the name of Smith in the United States.

The Bishop of Exeter has consecrated a new chapel at the South Devon and East Cornwall Hospital, Plymouth.

The Metropolitan Hospital, Kingsland Road, has received a cheque of £140, as the result of the Highbury Athenæum concert.

The Duke of Cambridge has promised to preside at the festival dinner, early in the coming year, of University College Hospital.

A ball, to benefit the Royal Eye Hospital, Southwark, was held last week at the Royal Institute of Painters in Water Colours.

The North-Eastern Hospital for Children is quite without funds, whilst the weekly average of little inmates is a really large one.

The Queen has subscribed 20 guineas to the Princess Louise Home, National Society for the Protection of Young Girls, Richmond Hill, Surrey.

Good work has been done at the Axminster Cottage Hospital during he past year, and we note a favourable balance on the funds of the institution.

The Queen has forwarded presents of cast linen to the East London Hospital for Children, Shadwell, and to the Royal Hospital for Incurables, West Hill, Putney Heath.

The annual report of the Royal Hospital for Incurables shows that the year has been exceptionally fruitful in legacies, the total amount realised from this source being £27,048.

Better results, financially, have been achieved at the Derbyshire Royal Infirmary, Derby, than in any previous year, and a balance of over £1,000 is existing now on the infirmary funds.

Princess Louise, Marchioness of Lorne, has forwarded a donation of £25 towards the funds of the Hospital for Children, Chelsea, of which hospital Her Royal Highness is patron.

A gift of 3,000 guineas has been received at the Middlesex Hospital from an anonymous donor as a perpetual endowment of one bed and two cots, in loving memory of his young wife.

The Prince of Wales has sent presents of game for the patients at Charing Cross and St. George's Hospitals, also to the Hospital for Epilepsy and Nervous Diseases, at Regent's Park.

The annual report of the Boston Hospital, Lincolnshire, shows an increase in the amount of subscriptions, patients' fees, &c. Over 200 patients have been treated during the year at the institution.

Mrs. Saxton, of the Elms, Shrewsbury, has transferred to the trustees of the Salop Infirmary, Shrewsbury, the sum of £2,000 for the endowment of two cots in the children's ward in memory of her parents.

The Royal Naval Sailors' Home, Queensferry, was benefited some time ago by a cheque of £1,000. It has since become known that the generous donor was Lord Rosebery, who has placed the home on a firmer financial footing through this gift.

The Prince of Wales has again sent a donation of 30 pheasants to the Royal Eye Hospital, Southwark. Attention is being called to the necessity for further funds for this most deserving charity in order to permit of the closed wards being reopened.

Additional accommodation is very seriously required at the Belfast Samaritan Hospital. Funds for this purpose would be a great boon, and the extension of premises would materially aid the surgeons in the discharge of their very arduous duties.

The Royal College of Physicians of Edinburgh has purchased property for the purposes of a laboratory. The premises, which are at the present time occupied, belong to the Royal Infirmary, and will soon be needed for the extension there required.

A very gratifying feature in the annual report of the Manchester Hospital for Diseases of the Ear is the amount the patients have themselves contributed towards its support; this has reached a sum about equal to that supplied by subscriptions and donations.

St. George's and St. James's Dispensary, in King Street, Regent Street, is seriously in need of funds if it is not to be closed. A comparatively small amount is required for the working of the charity, and it is much to be hoped that this will soon be forthcoming.

The National Food Supply Association is making an appeal on behalf of its funds. There are three large centres from which this most useful society turns out 12,000 meals a-day. The food is conveyed in tin carriers, through which means it arrives hot for the charge of one penny.

The sympathy of the public is being enlisted on behalf of the Royal Blind Pension Society, of 235, Southwark Bridge Road, London. By its charitable agency eight hundred deserving blind people in all parts of the kingdom are receiving regular assistance in their own homes, which in their desolate condition is greatly valued.

Dr. Garrod Thomas, J.P., has made a munificent offer of £5,000 to the Newport Infirmary, subject to the erection of the building in a different part of the town, the present one being unsuitable on sanitary and other grounds. We understand that the offer is conditional on another £15,000 being contributed by public subscriptions.

The Countess of Dudley, as president of the Birmingham and Midland Hospital for Women, is making an appeal on behalf of the charity, the subscription list of which is so small and the demands upon the institution so constantly on the increase that it has now become impossible to carry on the work without a very considerable increase in the revenue.

### THE STUDENT OF MEDICINE.

### APPOINTMENTS.

T. Beattie, M.B.Durh., B.S., appointed Pathologist to the Newcastle Infirmary. D. T. Belding, M.R.C.S.. L.R.C.P.Lond., appointed Medical Officer of Health to the Dereham District Council. W. C. T. Brown, B.A.Lond., M.B., Ch.B.Vict., late Medical Officer in charge of small-pox outbreak in district and town of East London, appointed to supervise the outbreak in Kimberley, Cape Colony. H. S. Byers, of the Northern Hospital, Liverpool, appointed House Surgeon of the Stockton and Thornaby Hospital. W. Calwell, M.A.Q.U.I., M.D.R.U.I., appointed Assistant Physician to the Belfast Royal Hospital. E. T. Chamberlain, L.R.C.P.Lond., M.R.C.S., appointed Medical Officer for the Banstead District of the Epsom Union. H. A. H. Claridge, M.B., B.S.Durh., appointed Medical Officer for the Norton Canes District of the Cannock Union. J. H. Dauber, M.A., M.B., B.Ch.Oxon., M.R.C.P., M.R.C.S., L.S.A., appointed Honorary Assistant Physicion to the Hospital for Women, Soho. G. M. Dawkins, L.R.C.P., M.R.C.S., appointed House Physician to the London Hospital. M. J. Doidge, B.A.Camb., M.R.C.S.Eng., appointed Medical Officer for the Fourth District of the Wells Union. W. H. Farmer, M.R.C.S., L.R.C.P., appointed Assistant House Surgeon to the Royal Portsmouth Hospital. A. Hardwick, M.D., appointed Medical Officer of Health to the Newquay Urban District Council. H. T. Heath, L.R.C.P.Edin., L.R.C.S.I., appointed Medical Officer of Health to the Mansfield Woodhouse Urban District. J. M. Hermon, M.B., C.M.Edin., appointed Medical Officer for the Sharlston District of the Wakefield Union. D. A. MacGregor, M.B., C.M.Edin., appointed Medical Officer of Health to the Skelmanthorpe Urban District Council. E. Middlebrooke, L.S.A., L.M., appointed Medical Officer to the St. Ives Union. J. Morgan, M.R.C.S.Eng., L.S.A., appointed Medical Officer for the Rheidol District of the Aberystwith Union. O. Norris, L.R.C.P.I., L.M., L.S.A., appointed Medical Officer of Health for the Sherburn Rural District. L. Potts, M.R.C.S.Eng., L.S.A., appointed Medical Officer for the Headley District of the Epsom Union. T. E. Rice, L.S.A., appointed House Surgeon and Dispenser to the Tiverton Infirmary. G. W. Roll, appointed Ophthalmic Surgeon to the Leicester Infirmary. C. E. Salter, M.D., B.S.Lond., F.R.C.S.Eng., appointed Honorary Medical Officer to the Scarborough Hospital and Dispensary. R. S. Smith, M.D.R.U.I., M.Ch., appointed Physician to the Belfast Royal Hospital. L. M. Snow, L.R.C.P.Lond., M.R.C.S.Eng., appointed Medical Officer for the No. 2 District of the Horsham Union. W. C. Steele, M.B., C.M., appointed Medical Officer for the Seventh District of the Tendring Union. D. S. Stephenson, appointed Medical Officer for the Woodstown Dispensary District. W. H. Vickery, F.R.C.S., appointed Surgical Registrar to the Royal Infirmary, Newcastle-on-Tyne. W. T. Wallington, L.S.A., appointed Medical Officer for the Bushbury District of the Cannock Union. C. C. Webb, M.B., B.C.Cantab, appointed Dispensary Surgeon to the Bradford Infirmary. R. M. West, M.R.C.S., L.R.C.P., appointed Surgeon to the Leicester Provident Dispensary. R. B. Wild, M.D.Lond., M.Sc.Vict., appointed Honorary Assistant Physician and Medical Superintendent of the Cancer Pavilion and Home, Manchester. S. Stephenson, M.B., M.Ch., F.R.C.S.E., appointed Ophthalmic Surgeon to the North-Eastern Hospital for Children, Shoreditch.

### VACANCIES.

The following vacancies are announced this week, the amount of salary in each case being given after the name of the institution:—

### Resident Medical Officers.

Bristol Incorporation.—Medical Officer for the Workhouse at Stapleton; doubly qualified. Salary £250 per annum, with residence and rates and taxes free, together with vaccination fees. Applications to J. J. Simpson, Clerk to the Guardians, St. Peter's Hospital, Bristol, by December 31st.

Derbyshire Royal Infirmary.—Resident House Surgeon and Resident House Physician; doubly qualified. Appointments tenable for twelve months, with a possibility of extension. Salaries £100 and £80 per annum respectively, with apartments and board. Applications, endorsed "House Surgeon" or "House Physician," to Walter G. Curnt, Secretary, by December 21st.

Devon County Asylum.—Assistant Medical Officer; single. Salary £120 per annum, with board, lodging, and washing. Applications to Arthur E. Ward, Clerk to the Visitors, 9, Bedford Circus, Exeter, by December 31st.

Devonshire Hospital, Buxton.—Assistant House Surgeon. Salary £50 per annum, with furnished apartments, board, and washing. Applications, endorsed "Assistant House Surgeon," to the Secretary by December 21st.

Liverpool Dispensaries.—Assistant Surgeon; unmarried. Salary £80, to be increased to £90 per annum after the first year's service, with apartments, board, and attendance. Applications to R. R. Greene, Secretary, 54, Moorfields, Liverpool, by December 23rd.

Poplar and Stepney Sick Asylum District.—Second Assistant Medical Officer for the Asylum at Bromley, Middlesex. Salary £80 per annum, increasing £10 yearly to £100. Applications, on forms provided, to be sent to Robert Foskett, Clerk to the Managers, Bromley, Middlesex, E., by January 2nd.

Taunton and Somerset Hospital.—Assistant House Surgeon. Appointment for six months, without salary, but board, washing, and lodging in the institution provided. Applications, endorsed "Assistant House Surgeon," to J. H. Biddulph Pinchard, Secretary, 13, Hammet Street, Taunton, by December 31st.

Westminster General Dispensary, Gerrard Street, Soho, W.—Resident Medical Officer. Applications to the Secretary by December 30th.

Wolverhampton and Staffordshire General Hospital, Wolverhampton.—Resident Assistant. Appointment for six months. Board, lodging, and washing provided. Applications, inscribed "Application for Resident Assistant," to the Chairman of the Medical Committee by December 30th.

### Non-Resident Medical Officers.

Dental Hospital for London, Leicester Square, W.C.—Assistant Dental Surgeon; must be L.D.S. Applications to J. Francis Pink, Secretary, by January 6th.

Dental Hospital for London and London School of Dental Surgery, Leicester Square, W.C. — Demonstrator. Honorarium £50 per annum. Applications to J. Francis Pink, Secretary, by January 6th.

### "THE HOSPITAL"

AN INDEPENDENT JOURNAL OF

**The Medical Sciences**

AND

**Hospital Administration,**

WITH WHICH IS ISSUED WEEKLY

**A SPECIAL**

### SUPPLEMENT FOR NURSES.

PUBLISHED EVERY SATURDAY.

PRICE TWOPENCE.

Matrons, Sisters, or Nurses will find in The Hospital a chronicle of the latest Nursing News, Practical Articles of the highest value to those who wish to keep themselves abreast with the times, and brightly written articles from Nurses all over the world.

**SUBSCRIPTIONS CAN COMMENCE AT ANY TIME.**

### TERMS OF SUBSCRIPTION.

### WEEKLY ISSUE.

| | Great Britain. | Foreign and Colonial. |
|---|---|---|
| For One Year ... ... | 10s. 6d. ... | 15s. 2d. |
| For Six Months ... | 5s. 3d. ... | 7s. 7d. |
| For Three Months... | 2s. 8d. ... | 3s. 10d. |

### MONTHLY PARTS.

| | Post Free to any Part of the World. |
|---|---|
| For One Year ... ... | 8s. 6d. |
| For Six Months ... ... | 4s. 3d. |
| For Three Months ... ... | 2s. 2d. |

Postal Orders and Cheques should be made payable to "The Scientific Press, Limited."

N.B.—In order to prevent delay, all business communications should be addressed to "The Manager" only, and not to any person individually.

### CHANGE OF ADDRESS.

In event of change of address, notice should be forwarded to the Publishers by the Saturday previous to date of publication, and the old address should also be given.

"The Hospital" can be obtained of all Booksellers and Newsagents in the United Kingdom, and at all the Railway Stalls, of Messrs. W. H. Smith and Son; and also from the following Agents in the Colonies and Abroad:—

Paris: Librairie Galignani, 224, Rue de Rivoli.

Berlin: Messrs. A. Asher and Co.

Leipsig: Mr. F. A. Brockhaus.

New York: The International News Co.

Montreal: The Montreal News Co.

Toronto: The Toronto News Co.

Melbourne, Sydney, Adelaide, and New Zealand: Messrs. Gordon and Gotch.

India: At all the Railway Bookstalls and Agencies of Messrs. A. H. Wheeler and Co.

South Africa: Messrs. Juta, Cape Town.

Publishing Offices: 428, Strand, London, W.C.

### NURSING: ITS THEORY AND PRACTICE,

BEING A

COMPLETE TEXT-BOOK OF MEDICAL, SURGICAL, AND MONTHLY NURSING.

By PERCY G. LEWIS, M.D., M.R.C.S., L.S.A., &c., &c.

To this edition have been added three chapters on Monthly Nursing, together with additions to the chapters on Blood Diseases, on Antiseptics, on the Nursing of Children, and on Private Nursing. Under these headings will be found a short account of the new Aseptic Treatment, the Antitoxin and Serum Treatments, and the Treatment by Thyroid Extracts.

These additions, combined with the revision throughout of the text, render this work the best and most complete Text-book on Nursing in the market, and strengthen its position as a Classic in the Training Schools of the World.

Third and Revised Edition. Demy 8vo., 209 pp., profusely Illustrated with Copyright Portraits and Drawings. Price 2s. 6d.

**HOW TO BECOME A NURSE:** And How to Succeed. A complete guide to the Nursing Profession for those who wish to become Nurses, and a useful book of Reference for Nurses who have completed their training and seek employment. Compiled by HONNOR MORTEN.

Second and Revised Edition. Demy 16mo., suitable for the apron pocket, handsomely bound in cloth, 140 pp. Price 2s.; in handsome leather, gilt, price 2s. 6d. net.

**THE NURSES' DICTIONARY OF MEDICAL TERMS AND NURSING TREATMENT.**—Compiled for the use of Nurses by HONNOR MORTEN. Containing descriptions of the principal Medical and Nursing Terms and Abbreviations, Instruments, Drugs, Diseases, Accidents, Treatments, Physiological Names, Operations, Foods, Appliances, &c., &c., encountered in the Ward or Sick-room.

Third Edition, enlarged and partly re-written, crown 8vo., cloth gilt, about 200 pp., price 2s. 6d.

**HOSPITAL SISTERS AND THEIR DUTIES.** By EVA C. E. LUCKES, Matron to the London Hospital. The favourable reception accorded to the first two editions of this useful and interesting work, and the regular demand for copies of it, encourage the publishers to place this third edition, greatly improved and enlarged, before the Institutions. The experience and position of its author entitle it to authority, and every Matron, Sister, or Nurse who aspires to become a Sister should read the advice and information contained in its pages.

Crown 8vo., 500 pp., Second Edition, 7 Plates, 18 Illustrations, Charts, &c., price 7s. 6d. net.

**NURSING.** By ISABEL ADAMS HAMPTON.

"It is not too much to say that in no single volume yet published has the subject been so scientifically, carefully, and completely treated as it has been by Miss Hampton. . . . Whether as a teaching manual or as a book of reference, the thoroughly practical instruction which it conveys is sure to obtain a wide circulation."—*The Hospital.*

Demy 8vo., cloth gilt, 260 pp. Price 3s. 6d.

**ART OF FEEDING THE INVALID.** By a MEDICAL PRACTITIONER and a LADY PROFESSOR OF COOKERY. A popular treatise on the most frequent disorders; with detailed lists of suitable diet and original recipes for daily use. This important work has been undertaken to supply a want long felt by Matrons and Lady Superintendents in Hospitals and Institutions of all kinds where invalids are treated, as well as in private households. It is of the greatest value to nurses in private practice.

Important New Handbook of Midwifery. Handsomely bound in leather, 260 pp., profusely illustrated, price 6s.

**A PRACTICAL HAND-BOOK OF MIDWIFERY.** By FRANCIS W. N. HAULTAIN, M.D. A Practical Manual produced in a portable and convenient form for reference.

LONDON: THE SCIENTIFIC PRESS (LIMITED), 428, STRAND, W.C.

### NURSING BOOKS AT FULL DISCOUNT.

*LIST POST FREE.*

The Theory and Practice of Nursing (Percy Lewis, M.D.), 3/6 for **2/8.**
The Nurse's Dictionary (Honnor Morten), 2/- for **1/6.**
The Nurse's Case Book, 6d. for **3½d.**

